# Supplementary material for: Comprehensive Two-Dimensional Gas Chromatography–Mass Spectrometry Analysis of Exhaled Breath Compounds after Whole Grain Diets
Source: Molecules. 2021 May 2;26(9):2667. doi: 10.3390/molecules26092667 (PMC8125105; doi:10.3390/molecules26092667)
Supplement: Supplementary file 1 [file molecules-26-02667-s001.zip › molecules-1183973-supplementary.pdf]

Table S1. The detected volatile organic compounds (86) with tentative identification[1] from exhaled breath samples (n=32) of nine study participants.

Compound name	RT	SI
e-1,3-Pentadiene	7.6	97
Carbon dioxide	7.6	95
z-1,3-Pentadiene	7.9	97
Isoprene	8.1	97
Pentane, 2,3-dimethyl	8.2	87
Dimethyl sulfide	8.4	94
1-Propene, 2-methyl-	8.4	91
Octane, 4,5-dimethyl	8.6	80
Hexane	8.7	96
Acetaldehyde	8.8	94
Heptane, 3,4-dimethyl-	8.8	87
Cyclopentane, methyl-	8.8	82
Heptane	9.0	87
Octane	9.5	89
Acetone	9.6	94
Ethyl acetate	9.9	87
3-Pentanol/ 2-propanol, 2-methyl	10.0	89
Butanal	10.3	92
2-Propanol	10.6	97
Propane, 1-(methylthio)-	10.7	85
2-Butanone	10.7	93
Ethanol	11.0	97
Methyl vinyl ketone	11.1	94
Sulfide, allyl methyl	11.2	85
Benzene	11.5	91
Pentanal	11.9	92
2-Pentanone	11.9	88
2,3-Butanedione	12.0	94
Methyl methacrylate	12.7	85
1-Propene, 1-(methylthio)-	12.9	91
α-Pinene	13.0	88
Acetonitrile	13.2	90
1-Propanol	13.8	94
Toluene	14.2	92
2-Butenal	14.3	91
Disulfide, dimethyl	14.8	84
Hexanal	15.5	95
Ethylbenzene	17.0	86
Benzene, 1,4-dimethyl-	17.5	91
1-Butanol	18.7	93
1,3,5-Trioxane	19.4	89
Benzene, 1,2-dimethyl-	19.5	89
Ethanol, 2-methoxy-	19.6	96

D-Limonene	19.9	96
Heptanal	20.0	94
Eucalyptol	20.3	89
Ethanol, 2-ethoxy-	21.3	95
Benzene, 1-ethyl-3-methyl-	21.4	82
1-Pentanol	23.0	82
Styrene	23.1	97
o/p-Cymene	23.4	82
Benzene, 1,3,5-trimethyl-	24.0	87
Cyclohexanone	24.4	84
Octanal	24.6	96
Undecane	25.2	86
α-Methylstyrene	26.7	88
5-Hepten-2-one, 6-methyl-	27.0	93
Ethanol, 2-(ethenyloxy)-	28.2	94
Nonanal	29.5	97
Tetradecane	30.1	84
Benzoic acid [2]	30-69	95
2-Decen-1-ol	32.1	87
1-Hexanol, 2-ethyl-	34.0	96
Decanal	34.6	95
1,3,5,7-Tetroxane	35.6	82
Benzaldehyde	36.0	89
Acetophenone	40.7	89
Cyclohexanol, 5-methyl-2-(1-methylethyl)-	40.7	90
Benzaldehyde, 4-methyl- / Benzaldehyde, 2-methyl-	40.8	87
Ethanol, 2-(2-ethoxyethoxy)-	40.8	95
Phthalic acid / Phthalic anhydride [2]	40-69	93
Anethole	47.8	90
Propanoic acid, 2-methyl-, 1-(1,1-dimethylethyl)-2-methyl-1,3-propanediyl ester	49.4	80
Hexanoic acid	50-60	85
Phenol	53.9	90
2(3H)-Furanone, dihydro-4-hydroxy [2]	57.5	85
Ethanol, 2-phenoxy	58.8	90
ethanedione, diphenyl [2]	61.7	94
3,6,9,12-Tetraoxatetradecan-1-ol	62-69	88
2(3H)-Furanone, 5-dodecyldihydro- [2]	63.7	92
1-Dodecanol, 2-octyl-	64.8	93
Benzamide [2]	65.5	94
2(3H)-Furanone, dihydro-5-tetradecyl [2]	67.7	89
Phenol, 2,4-bis(1,1-dimethylethyl)-	67.8	92
4-Nonylphenol	67.9	90
Methanone, diphenyl	69.1	70

RT = retention time (min), SI = similarity index in the mass spectrum comparison, [1] tentative identification was made by comparing mass spectrum with mass spectral databases and not confirmed by using retention indices

or analytical standards, compounds detected only in a single exhaled breath sample were excluded, [2] detected only during whole grain diet
